# Effect of Comedications and Endotoxins on Mesenchymal Stem Cell Secretomes, Migratory and Immunomodulatory Capacity

**DOI:** 10.3390/jcm8040497

**Published:** 2019-04-11

**Authors:** Nisha Durand, Athena Russell, Abba C. Zubair

**Affiliations:** Transfusion Medicine, Department of Laboratory Medicine and Pathology and Center for Regenerative Medicine, Mayo Clinic, Jacksonville, FL 32224, USA; durand.nisha@mayo.edu (N.D.); athena.liza.russell@emory.edu (A.R.)

**Keywords:** mesenchymal stem cells, immunosuppressive drugs, LPS, secretome, cytokines, cell migration, immunomodulation

## Abstract

Mesenchymal stem cells (MSCs) are becoming an increasingly popular therapeutic option among patients with a broad range of ailments to modulate immunity and induce regeneration. The majority of patients receiving these MSC therapies are on concurrent medication or have ongoing infection. In the present study, we examined the effect of immunosuppressive drugs and lipopolysaccharides (LPS)/endotoxins on the secretory profile, migration towards site of injury, and suppression of lymphocyte proliferation of bone marrow-derived MSCs (BMSCs). Generally, LPS coculture augmented the secretory capacity of BMSCs while exposure to immunosuppressive drugs resulted primarily in no change or attenuated secretion, with some cases of increased secretion, dependent on the cytokine assayed. Among the immunosuppressants evaluated, Hydrocortisone had the most widespread inhibitory effect, while LPS from *E. coli* O111:B4 had the most potent stimulatory effect. In addition, we also showed that Hydrocortisone or LPS from *E. coli* O111:B4 affected the migratory and immunosuppressive capacity of BMSCs. Following simulation with Hydrocortisone, BMSC migration was attenuated, and immunosuppressive capacity against T cell proliferation was enhanced, however, the opposite effects were seen with LPS from *E. coli* O111:B4. Our data suggests that the clinical outcomes of MSC-based therapy are affected by the use of immunosuppressive medication or the presence of endotoxemia in patients.

## 1. Introduction

Mesenchymal Stem Cells (MSCs), the best characterized adult stem cells, are defined by the expression of specific markers, namely CD105, CD73, and CD90, lack of expression of CD45, CD34, CD11b, CD19, and HLA-DR surface receptors, and their ability to give rise to osteoblasts, adipocytes, and chondrocytes in vitro [1,2,3]. MSCs secrete a broad range of chemokines, cytokines, and growth factors [3,4], and have been isolated from multiple organs and tissues including bone marrow, adipose tissue [5], endometrium [6], periodontal ligament, dental pulp [7], and synovial fluid [8]. The self-renewing, immunomodulatory, differentiation, and secretory capacity of MSCs make them an attractive therapeutic agent. Indeed, MSCs have been the subject of countless preclinical studies and clinical trials [9,10], where their mechanism of action, safety and efficacy have been evaluated in a wide range of aliments, from neurological disorders [11] to transplantation-associated rejection and morbility [12,13]. Despite the many completed or ongoing clinical trials involving MSCs [14], to date, only a limited number of MSC-based products are currently approved for clinical use.

In the future, persons suffering from immunological derangement could stand to benefit the most from MSC-based therapies, and according to several reports, the utility of MSCs in this setting is a direct consequence of their potent immunomodulatory activities [15,16,17]. Seminal studies by Di Nicola et al. showed that MSCs derived from bone marrow (BM) could inhibit T cell proliferation in a manner which was dependent on transforming growth factor beta1 (TGFβ1) and hepatocyte growth factor (HGF) secretion [18]. MSCs are recruited to sites of active inflammation or tissue injury where they secrete anti-inflammatory molecules like interleukin-10 (IL-10), HGF, TGFβ1 [19], and indoleamine 2,3-dioxygenase (IDO) [20]. In addition to secreting these regulatory molecules themselves, MSCs also influence the secretory profile of other cells to promote and maintain an anti-inflammatory environment [21,22]. For example, in response to IL-12 and IL-18 stimulation, MSCs can prime natural killer (NK) cells for enhanced release of interferon-γ (INF-γ) [23], a cytokine which exhibits both pro- and anti-inflammatory properties [24,25]. While MSCs seem like a feasible and promising therapeutic option for patients, especially those with immune-related diseases such as graft vs. host diseases (GVHD), a proportion of these patients are likely to be on some type of immunosuppressive regimen [26]. The function of MSCs could be influenced by drugs designed to attenuate immune function and agents such as liposaccharides (LPS)/endotoxins that can augment inflammatory responses [27].

In this study, we sought to determine the effect of immune function modulators: immunosuppressive medications to include Cyclosporin, Tacrolimus, Hydrocortisone, Dexamethasone, Rapamycin and Mycophenolic acid (MPA) or LPS derived from two different *E. coli* strains (O111: B4 and O26:B6) on the secretory profile of bone marrow-derived mesenchymal stem cells (BMSCs). Our results indicate that the secretomes of BMSCs which regulate activation, regenerative and anti-inflammatory function, are highly responsive to cues that can either elicit or attenuate immune responses. Stimulation with Hydrocortisone had the most potent inhibitory effect on the secretory profile of BMSCs, while LPS from *E. coli* O111: B4 had the most potent stimulatory effect. In addition, we show that BMSC migratory capacity and their ability to inhibit T cell proliferation is altered in the presence of Hydrocortisone and LPS from *E. coli* O111:B4.

## 2. Materials and Methods

### 2.1. Primary Cell Cultures, Antibodies, and Reagents

MSCs (BMSC22, BMSC32, and BMSC34) were obtained from commercially sourced bone marrow (Lonza, Basel, Switzerland) from three independent donors. Each bone marrow was combined with PBS (1:3 ratio), layered on top of Ficoll-Paque PREMIUM 1.077 (GE Healthcare, Chicago, IL, USA) (4:3 ratio) and centrifuged for 40 min at 400× *g*. The Mononuclear Cell (MNC) layer was isolated, washed with PBS, and residual red blood cells were lysed with ACK lysis buffer. After MNC number was determined using a BD Accuri C6 Flow Cytometer, (Becton Dickinson: (Franklin Lakes, NJ, USA). MNCs were subsequently seeded to T1000 culture flasks at a density of 100,000 MNCs/cm^2^ in a αMEM supplemented with 5% human platelet lysate and 1% GlutaMAX-I (Complete Medium).Complete medium was replenished every 3–4 days until MSC colonies were about 90% confluent. Cells at P0 were passaged, and seeded at P1 to T1000 culture flasks at a density of 5000/cm^2^. Fresh complete medium was replenished every 3–4 days. BMSCs were harvested when cell monolayers reached about 90% confluency and frozen in cyogenic vials (2.0 × 10^6^ or 2.4 × 10^6^ cells) to generate Master Cell Banks (MCB). MCB vials were retrieved to perform further experimental analysis as described below, and all experiments were performed in cells between P1 and P5.

PBMCs were from Astarte Biologics (Bothell, WA, USA). Cyclosporin, Tacrolimus, Rapamycin were from InvivoGen (San Diego, CA, USA) and Mycophenolic acid, Dexamethasone, Hydrocortisone and Lipopolysaccharides were from Sigma (St. Louis, MO, USA). Antibodies: CD90-PE, CD105-PE, CD73-PE, CD45-PE, CD34-PE, CD11b-PE, CD11b-PE, CD19-PE and HLA-DR-PE were all from Becton Dickinson (Franklin Lakes, NJ, USA). PHA-P and DAPI were from Sigma and Paraformaldehyde was from Biotium Inc (Fremont, CA, USA). PBS, ACK lysis buffer, αMEM, GlutaMAX-l was from Thermo Fisher Scientific (Waltham, MA, USA), Ficoll-Paque PREMIUM 1.077 from GE Healthcare (Chicago, IL, USA) and human platelet lysate from Cook Regentec (Indianapolis, IN, USA).

### 2.2. Flow Cytometry

BMSCs at P1 were incubated with the primary antibodies listed above and flow cytometry analysis was performed using a BD Accuri C6 Flow Cytometer to quantify the expression of positive and negative MSC surface markers as defined by the ISCT.

### 2.3. Drug-Induced Cytokine Secretion

BMSCs at passage 2 were seeded in 6-well plates at 0.5 × 10^5^ MSC per well in αMEM complete medium and allowed to adhere overnight. The following day, dilutions were made of complete medium containing each drug or LPS in the following concentrations; Cyclosporin (10, 100, 500 ng/mL), Mycophenolic acid (1, 10, 100 µg/mL), Tacrolimus (1, 10, 100 ng/mL), Rapamycin (1, 10, 50 ng/mL), Dexamethasone (0.01, 0.1, 1.0 ng/mL), Hydrocortisone (1.0, 4.0, 8.0 µM), LPS from *E. coli* O111:B4 and O26:B6 (1, 5, 10 µg/mL). αMEM complete medium was changed to the drug/LPS media, and MSCs were incubated at 37 °C/5% CO_2_ for 72 h. Conditioned medium was collected at the end of the incubation period and stored at −80 °C until cytokine analysis was performed. Conditioned medium was analyzed for the presence and concentration of 42 different cytokines and growth factors using the Human Cytokine Array/Chemokine Array 42-plex Discovery Assay® (MilliporeSigma: Burlington, MA, USA) and performed by Eve Technologies (Calgary, AB, Canada).

The 42-plex assay included epidermal growth factor (EGF), eotaxin-1, fibroblast growth factor-2 (FGF-2), Flt-3L, Fractalkine, granulocyte–colony-stimulating factor (G-CSF), granulocyte macrophage colony stimulating factor (GM-CSF), GRO(pan), interferon (IFN)-α2, IFN-ƴ, interleukin(IL)-1a, IL-1b, IL-1ra, IL-2, IL-3, IL-4, IL-5, IL-6, IL-7, IL-8,IL-9, IL-10, IL-12 (p40), IL-12 (p70), IL-13, IL-15, IL-17A, IL-18, IFN-ƴ inducible protein (IP)-10, monocyte chemoattractant protein-1 (MCP-1), MCP-3, macrophage-derived chemokine (MDC), macrophage inflammatory protein (MIP)-1α, MIP-1β, platelet-derived growth factor AA (PDGF-AA), PDGF-BB, RANTES, sCD40L, transforming growth factor (TGF)-α, tumor necrosis factor (TNF)-α, TNF-β, and vascular endothelial growth factor A (VEGF-A). Red indicates increased cytokine secretion and green represents reduced secretion. Intensities indicate fold changes.

### 2.4. Cell Migration Assay

Migration assays were conducted using 8 µM ThinCert Transwells (USA Scientific, Ocala, FL, USA). BMSCs at Passage 4 were seeded in triplicate at a density of 1 × 10^5^ cells/transwell in αMEM complete medium containing hydrocortisone (0, 1.0, 4.0, 8.0 µM) or LPS from *E. coli* O111:B4 (0, 1, 5, 10 µg/mL). Conditioned medium collected from PBMCs previously stimulated with PHA-P (4 µg/mL) was added to the bottom of the transwell to induce cell migration. After 24 h, cells on the top of the transwell were removed and cells that had migrated to the lower surface were fixed with 4% PFA/PBS for 30 min (room temperature) and stained with DAPI. Images of the DAPI-positive cells were captured using the EVOS FL Imaging System (Thermo Fisher Scientific: Waltham, MA, USA) and subsequently manually quantified using Adobe Acrobat’s Counting Tool. Cell migration was normalized to cell numbers obtained from control wells containing αMEM complete medium only.

### 2.5. T Cell Proliferation Assays

BMSCs at P4 were seeded to 24 well plates at a density of 75,000 cells/well in αMEM complete medium containing hydrocortisone (1.0, 4.0, 8.0 µM) or LPS from *E. coli* O111:B4 (1.0, 5.0, 10.0 µg/mL). PBMCs were added to each of the wells at a density of 300,000 cells/well. This represents a sequence of wells containing BMSC: PBMC at a fixed ratio of 1:4, with increasing doses of Hydrocortisone or LPS from *E. coli* O111:B4. Control wells containing PBMCs only and BMSC-PBMC only with no hydrocortisone or LPS were also included. PHA-P (5 µg/mL) was added to wells to induce T cell activation. The assay plate was placed in a 37 °C, 5% CO_2_ incubator for 72 h, at which time the cells were harvested and stained with anti-CD3 and anti-CD45 antibodies and subsequently analyzed using an Accuri C6 Flow Cytometer for T cell enumeration.

### 2.6. Statistical Analysis

Data are represented as mean ± SD. p values were determined using GraphPad Prism’s (GraphPad Software, San Diego, CA, USA) One-way ANOVA test, and statistical significance was considered as *p* < 0.05.

## 3. Results

### 3.1. The Cytokine Secretion Profile of Bone Marrow Derived Mesenchymal Stem Cells is Influenced by Immunosuppressive Medications and LPS

**Figure 1:**
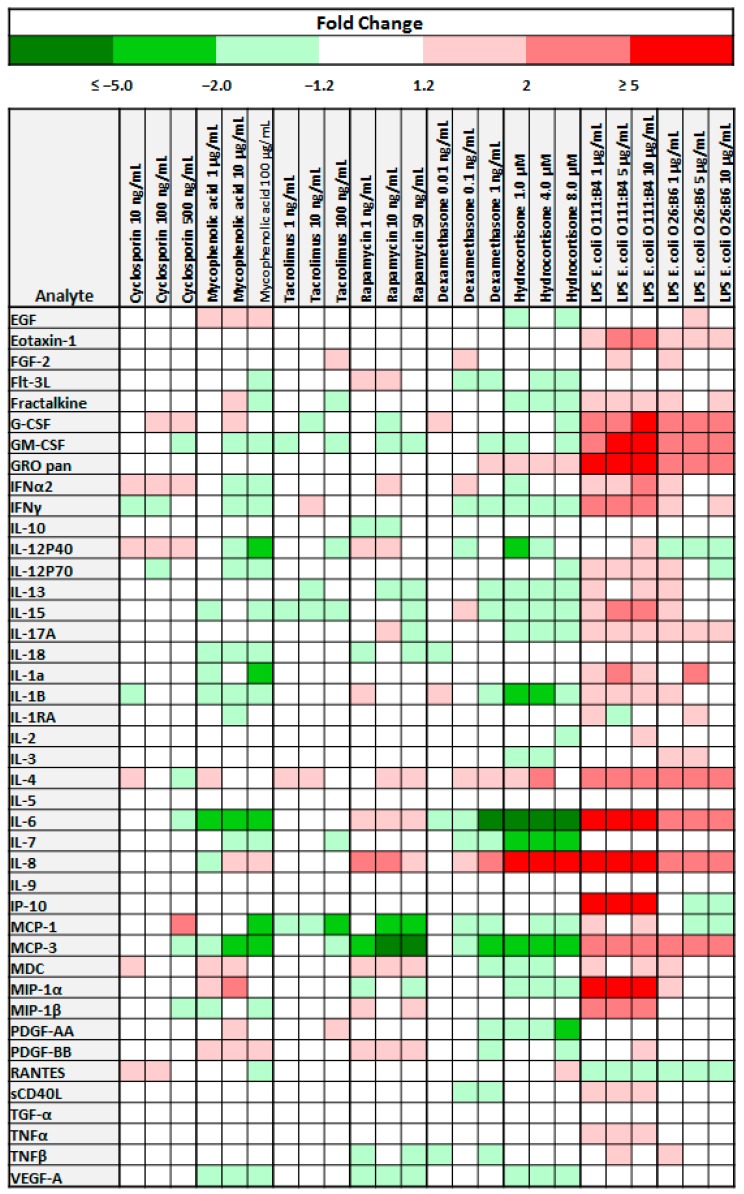
Cytokine secretion profiles for bone marrow-derived MSCs (BMSCs) treated with immunosuppressive medications or lipopolysaccharides (LPS). Heat Map of combined fold change in cytokine secretion for three cell lines (BMSC22, BMSC32 and BMSC34) after 3 days of coculture with immunosuppressants or LPS. EGF—epidermal growth factor, FGF-2—fibroblast growth factor-2, Flt3L—Fms-related tyrosine kinase 3 ligand, G-CSF—granulocyte–colony-stimulating factor, GM-CSF—granulocyte macrophage colony stimulating factor, IFN—interferon, IL—interleukin, MCP—monocyte chemoattractant protein, MDC—macrophage-derived chemokine, MIP—macrophage inflammatory protein, PDGF—platelet-derived growth factor, TGF—transforming growth factor, TNF—tumor necrosis factor, VEGF—vascular endothelial growth factor.

Conditioned medium from three independent primary cell lines; BMSC22, BMSC32, and BMSC34 (Morphological and immunophenotypic characterization of cell lines provided in Supplemental Figure S1) stimulated with immunosuppressants or LPS were evaluated for the presence and concentration of 42 secreted cytokines and chemokines. Fold changes in analyte concentration in response to stimulation with immunosuppressants or LPS, in relation to control for each drug/LPS dilution is depicted by the Heat Map in Figure 1.

Overall, we found that both immunosuppressive drugs and LPS have distinct effects on the secretomes of BMSCs (Figure 1). For many of the immunosuppressants, compared to control samples, there were no significant differences in the level of cytokines/chemokines secreted. When stimulation with the drugs resulted in changes in cytokine secretion, though both increased and attenuated secretion was observed, the latter was more widespread. The response to immunosuppressive drugs was quite variable; not only as it relates to the specific drug but also in response to the specific dosage at which the drug was applied. For example, while all doses of MPA led to increased secretion of EGF, reduced secretion of IL-18, IL-1B, IL-6, and VEGF-A was observed. IL-4 provides an example of a cytokine that exhibited a varied secretion pattern in response to the same immunosuppressive agent (cyclosporin).

Analytes particularly important for MSC function, such as IL-6, IFNγ, and TNFα, also exhibited a varied secretory pattern. There were no significant changes in TNFα secretion for any of the six immunosuppressants evaluated.

For IL-6, with Hydrocortisone, there was at least a 5-fold reduction in its secretion, at least 2-fold reduction with MPA, and at least a 1.2 fold increase with Rapamycin. On the other hand, decreased secretion of IFNγ was noted for four (Cyclosporin A, MPA, Dexamethasone, Hydrocortisone) out of the six drug evaluated, when applied at specific concentrations. There were also instances when stimulation with immunosuppressive medications led to increased secretion of specific cytokines; for example, treatment with Rapamycin increased the secretion of IL-8, MDC and PDFG-BB and stimulation with MPA resulted in increased secretion of EGF and PDGF-BB. Among all the immunosuppressants examined, Hydrocortisone had the most wide-spread inhibitory effect on cytokine secretion.

For LPS stimulation, we found that the secretion of most cytokines was enhanced or remained unchanged (Figure 1). There were a few instances in which a decrease in analyte concentration was noted. Though when compared to LPS from *E. coli* O26:B6, LPS from *E. coli* O111:B4 had a greater effect on cytokine secretion, there was more uniformity in the response patterns seen between the LPS as compared to the immunosuppressants. For example, at all three concentrations, stimulation with either LPS increased the concentration of Eotaxin-1, G-CSF, GM-CSF, GRO pan, IL-17A, IL-4, IL-6, IL-8, and MCP-3. Of note, for GRO pan, IL-6, IL-8, IP-10, and MIP-1α, there was at least a 5-fold increase in secretion following stimulation with LPS from *E. coli* O111:B4. Generally, the secretory capacity of BMSCs was enhanced by LPS stimulation.

As depicted in (Figure 1), the cytokine secretion profile of BMSCs has a wide-ranging response pattern in response to stimulation with immunosuppressants or LPS; with Hydrocortisone having the most significant inhibitory effect and LPS from *E. coli* O111:B4 having the most potent stimulatory effect.

### 3.2. Stimulation with Hydrocortisone or LPS from E. coli O111:B4 Regulates BMSC Migration

**Figure 2:**
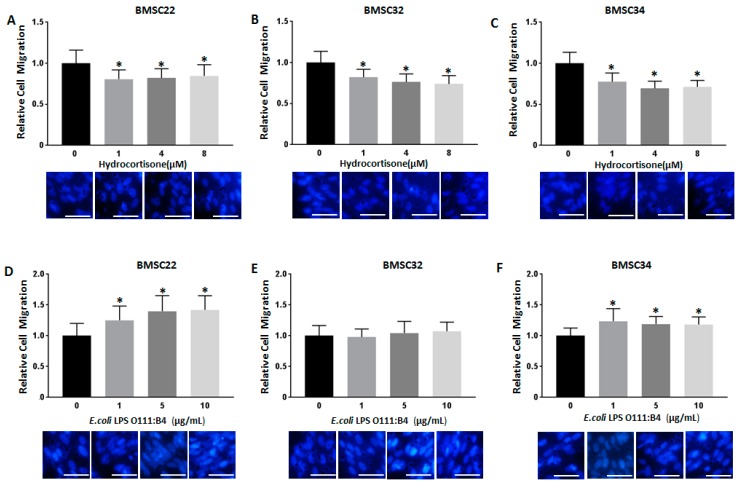
Stimulation with Hydrocortisone or *E. coli* O111:B4 regulates BMSC migration. The migration capacity of BMSC22, BMSC32 and BMSC34 in presence of Hydrocortisone (**A–C**) or LPS (**D–F**) was evaluated by Transwell Assays. BMSCs were seeded on top of Transwells, and after 24 h the cells that had migrated to the lower chamber were fixed, stained and quantified. Representative images of cells stained with DAPI are depicted below. (Scale bar is 50 µM, Magnification is 40×). * *p* < 0.05 versus 0 µM Hydrocortisone or 0 µg/mL LPS as determined by one-way ANOVA.

Since the cytokine secretion profile of BMSCs was responsive to stimulation with both immunosuppressants and LPS, we decided to examine the effect of the most potent immunosuppressant and LPS on directed cell migration. In vivo, MSCs home to sites of injury [28] and inflammation [29], so we evaluated their capacity to migrate to conditioned medium from Peripheral Blood Mononuclear Cells (PBMCs) stimulated with phytohemagglutinin-p (PHA-P), a T cell mitogen (Figure 2A–C).

We found that for all three primary cell cultures (BMSC22, BMSC32, and BMSC34), stimulation with Hydrocortisone at all doses resulted in modest, but significant decreases in cell migration when compared to control samples. For BMSC22, on average, cell migration was reduced by 18% for all doses of Hydrocortisone (Figure 2A), for BMSC32, 23% (Figure 2B), and for BMSC34, 28% (Figure 2C). The decrease in cell migration was dose-dependent for BMSC32, but for BMSC22 and BMSC34, this dose effect was not observed.

Simulation with *E. coli* LPS O111:B4 had a somewhat opposite effect on BMSC migration than the effect produced by Hydrocortisone (Figure 2D–F). Increased migration was noted for BMSC22 and BMSC34, while for BMSC32 there were no significant changes in cell migration. Treatment with LPS O111:B4 resulted in a dose-dependent increase in cell migration for BMSC22, which on average represented about a 35% increase (Figure 2D). Since there was less than 10% increase in migration for BMSC32 with LPS at all doses, these changes were not deemed significant (Figure 2E). With BMSC34, there was an overall average increase in cell migration of about 20% for all LPS doses applied; however, this effect was not dose-dependent (Figure 2F).

Collectively, this data demonstrates that migration of BMSCs towards a specific stimulus is generally attenuated when these cells are cocultured with Hydrocortisone but augmented when cocultured with LPS from *E. coli* O111:B4.

### 3.3. The Immunosuppressive Capacity of BMSCs is Regulated by Hydrocortisone and LPS from E. coli O111:B4

**Figure 3:**
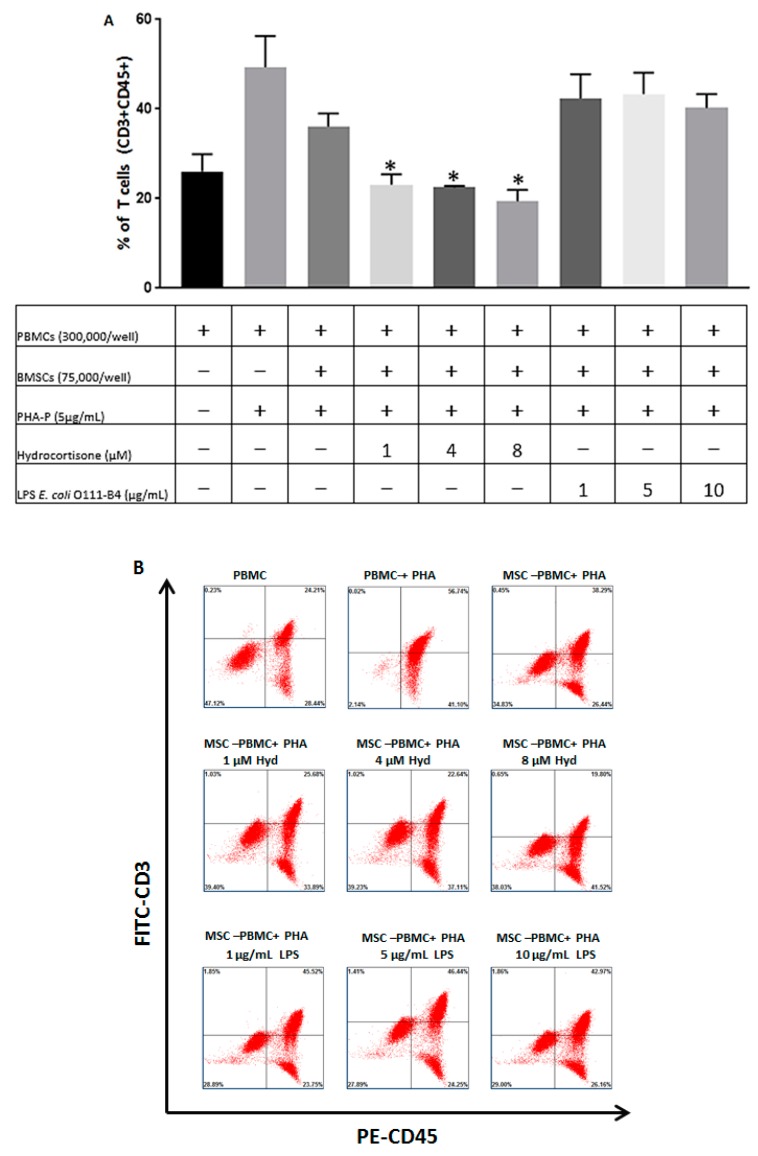

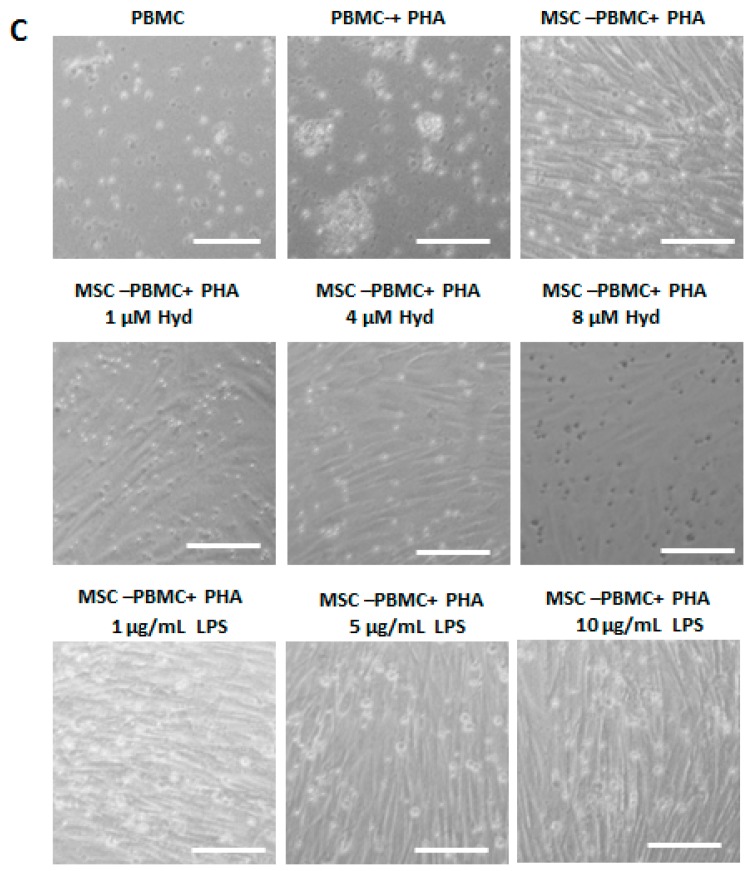
Stimulation with Hydrocortisone or *E. coli* O111:B4 affects BMSC immunosuppressive function. (**A**) Percentage of T-cells was determined by flow cytometry after coculture with BMSCs in the presence of Hydrocortisone or LPS from *E. coli* O111-B4. The results of statistical analysis for the percentage of CD3+CD45+ T cells are depicted as the mean ± S.D for the three cell lines (BMSC22, BMSC32 and BMSC34) evaluated. (**B**) Representative example of gating strategy for determination of T cell population after coculture with BMSC22 in presence of Hydrocortisone or LPS from *E. coli* O111-B4. (**C**) Representative images of BMSC22 and PBMC coculture in presence of Hydrocortisone or LPS from *E. coli* O111-B4. Scale bar: 50 µm. * *p* < 0.05 versus MSC-PBMC only (3rd column), as determined by one-way ANOVA.

Having shown that BMSC migration can be regulated by exposure to hydrocortisone and LPS, we sought to determine if the function of BMSCs once at their target site could likewise be influenced by the aforementioned agents. Figure 3A depicts flow cytometry results for the percentage of T cells (CD3+CD45+) after coculture with BMSCs in presence of hydrocortisone or LPS from *E. coli* O111:B4. As expected, we found that naïve BMSCs (no Hydrocortisone or LPS stimulation), when cocultured with PBMCs in presence of PHA-P, even at a BMSC-PBMC ratio of 1:4, resulted in a significant decrease in T cell proliferation (compare column 2 with column 3).

In presence of Hydrocortisone, the inhibitory effect of BMSCs on T cell proliferation was augmented. For all three doses of hydrocortisone, there was an even further reduction in the percentage of T cells, as compared to the coculture of BMSC-PBMC in the absence of Hydrocortisone, and this is a partial dose-dependent effect (Figure 3A). On the other hand, LPS had the opposite effect of Hydrocortisone on the percentage of T cells. Our data indicates that LPS, when applied at all three doses, reversed the effect of BMSCs on T cell proliferation, in a manner which is not dose dependent. The representative gating strategy for BMSC22 used for quantifying the percentage of T cells shown is Figure 3A is depicted in Figure 3B. Representative coculture images for BMSC22 and PBMCs are illustrated in Figure 3C. As shown in Figure 3C, in presence of PHA-P and absence of BMSCs, PBMCs are the most abundant. After the addition of BMSCs, the number of PBMCs is reduced, and the introduction of Hydrocortisone resulted in a further reduction in the number of PBMCs. When LPS from *E. coli* O111-B4 is introduced, the number of PBMCs present is increased as compared to the MSC-PBMC control. In response to stimulation with Hydrocortisone and LPS from *E. coli* O111-B4, the BMSCs themselves also exhibited some morphological changes, but nonetheless, overall, this data demonstrates that in presence of Hydrocortisone, BMSCs exhibit enhanced ability to inhibit T cell proliferation while the phenomenon is reversed in presence of LPS from *E. coli* O111:B4.

## 4. Discussion

There has been a long-standing interest in understanding the mechanisms that govern cytokine and growth factor secretion in BMSCs since this is the means by which these cells are thought to mediate their therapeutic effects. BMSC function has already been shown to be modulated in the presence of immunosuppressants as was demonstrated by Popp et al, who showed that the efficacy of BMSCs in inducing long-term acceptance of solid transplant allografts was enhanced in combination with low-dose mycophenolate in a rat heart transplantation model [30]. As we have shown in the current study, immunosuppressants like Mycophenolic acid (MPA) produce distinct effects on the cytokine secretion profile of BMSCs, and it likely that these changes contribute to the altered function of BMSCs, as reported in the study above.

Our data shows that the change in cytokine secretion of BMSCs when stimulated with various immunosuppressants is quite varied. This variability could be attributed to multiple factors. The heat map presented in Figure 1, collectively represents three independent BMSC donor-derived primary cell lines (BMSC22, BMSC32, and BMSC34), and it is known that BMSCs isolated from different donors exhibit functional variability. Recently published data from our own lab indicates that BMSC physiology is significantly impacted by donor-to-donor variability [31]. The varied cytokine response could also be attributed to the specific mechanisms of action of the immunosuppressants assayed. In our study, the drugs we used included Calcineurin inhibitors like Cyclosporin and Tacrolimus [32] and steroid-derived immunosuppressants like dexamethasone and hydrocortisone [33]. A different response pattern to medications in the same class is not unique to our study. In a previous study, Rapamycin and calcineurin inhibitors antagonized the inhibitory of effect of BMSCs on lymphocyte proliferation, while MPA promoted it and Dexamethasone did not have any effect [34].

While all the drugs assayed in this study are used to promote immunosuppression, our data clearly indicates that the secretomes of BMSCs were differentially affected by each respective immunosuppressive medication. This information could be of potential importance in clinical settings. We have previously shown that neuronal recovery induced by BMSCs is in part mediated by VEGF-A secretion [35], and as such immunosuppressants that modulate VEGF-A levels could potentially affect clinical outcomes. For example, if there were a patient population for which the intended function of BMSC therapy was to induce neuronal regeneration & recovery, this effect might be compromised in patients who are medicated with MPA, Rapamycin or Hydrocortisone since these agents inhibit VEGF-A secretion.

Unlike the variability seen with immunosuppressive medications, the secretory pattern of BMSCs in response to LPS stimulation was a bit more consistent. Endotoxins are known to induce cytokine secretion and activation of multiple immune cells, for example, LPS derived from *P. gingivalis* or *E. coli* can induce the release of TNF, VEGF, and MCP-1 from mast cells [36] and experiments conducted in C57BL/6 mice showed that *P. gingivalis*-derived LPS induced NK cell proliferation and activation in vivo [37]. Given that LPS are derived from bacteria, it is not too surprising that stimulation with LPS resulted in enhanced secretion of proinflammatory cytokines like G-CSF, GM-CSF and MCP-3. Increased secretion of proinflammatory cytokines could serve as a potential mechanism for eliciting an immune response and subsequent clearance of bacterial pathogens.

With the changes in the secretome of BMSCs resulting from stimulation with either immunosuppressants or LPS, we explored in further detail how these changes affected specific BMSC functions. Since Hydrocortisone has the most inhibitory effect and LPS from *E. coli* O111:B4 had the most potent stimulatory effect, we used these two agents as a surrogate for evaluating BMSC function during suppressed and heightened inflammatory states, respectively. We initially examined the effect of hydrocortisone and LPS from *E. coli* O111:B4 on BMSC migration towards PBMC conditioned medium. Stimulation with all doses of Hydrocortisone attenuated BMSC migration (Figure 2A–C), consistent with previous reports where Hydrocortisone was shown to impede migration in other cell types [38]. Inhibition of BMSC migration could be due to reduced expression of cytokines such as IL-6, which BMSCs not only secrete but can also respond to [39], and which has been shown to positively regulate cell migration via MMP-9 [40]. Likewise, the LPS O111:B4-induced cell migration seen in BMSC22 and BMSC34 (Figure 2D,F) can also be attributed to heightened secretion of factors like TNFα, which was recently shown to increase dental pulp stem cell migration via integrin upregulation [41]. The fact that stimulation with LPS O111:B4 did not significantly affect the migration of BMSC32 (Figure 2E) further validates the notion that BMSCs derived from different donors exhibit significant functional variability.

Having shown that BMSC migration is modulated in the presence of Hydrocortisone and LPS from *E. coli* O111:B4, we expected a change in the immunosuppressive capacity of BMSCs when cocultured with Hydrocortisone or LPS. We found that in the presence of Hydrocortisone, the ability of BMSCs to inhibit T cell proliferation was augmented (Figure 3A). As an immunosuppressive agent, hydrocortisone dampens the inflammatory response and the associated T cell proliferation, and consequently in this setting, the inhibitory effect of BMSCs is potentiated. As we have shown for hydrocortisone in the current study, similar effects of other immunosuppressants have been established as was demonstrated in one study, where the inhibitory effect of MSCs on natural killer (NK) activation was reported to be augmented by dexamethasone [42]. Our data indicates that stimulation with LPS from *E. coli* O111:B4 resulted in attenuation of BMSCs’ immunosuppressive capacity (Figure 3A). Stimulation with LPS induced secretion of known T cell activators such as INFƴ [43] therefore, in this setting, the LPS effect is more on T cell activation than BMSC stimulation. As a result the BMSCs would have to counteract this heightened activation state of immune cells and subsequently their immunosuppressive capacity is diminished. Conversely, the potentiated effect of BMSCs immunosuppressive capacity towards PBMCs when cocultured with hydrocortisone can also be attributed to the attenuated secretion of INFƴ in response to hydrocortisone stimulation.

For the cell migration and BMSC-PBMC coculture assays, even though we evaluated LPS from only one *E. coli* Strain (O111:B4), we would expect LPS from *E. coli* O26:B6 to have a similar effect on BMSC migration and modulation of T cell proliferation, since the secretion pattern of BMSCs in response to both LPS has a high degree of overlap. Though we used hydrocortisone as a surrogate for attenuated inflammatory states induced by immunosuppressants, it is clear that the drugs we evaluated in the cytokine assay have distinct effects on BMSC secretion (Figure 1). It is plausible that the effect on BMSC migration and regulation of T cell proliferation seen with Hydrocortisone might not be concordant with the other drugs, like Rapamycin or Tacrolimus. However, we believe that the data obtained from the migration and coculture assays obtained with Hydrocortisone still gives insight into MSC function in dampened inflammatory states, and while evaluating the effect of each immunosuppressant on BMSC migration and modulation of T cell proliferation was beyond the scope of this study, it does warrant further investigation in future studies.

Overall, in this study we showed that the secretomes of BMSCs can be influenced by immunosuppressive medications or LPS, and that this change in cytokine secretion is also associated with alterations in key BMSC functions such as cell migration and immunomodulation. In response to Hydrocortisone stimulation, BMSC migration was diminished but immunosuppressive capacity against T cells proliferation was augmented; the converse effect was seen following stimulation from LPS from *E. coli* O111:B4. Our data also demonstrates that BMSCs exhibit variability in their response pattern to various stimuli. As depicted by our findings, BMSCs are highly responsive to cues such as immunosuppressive medications or LPS, and as such the presence of these factors in certain study cohorts should be accounted for when these patients are subjected to MSC-based therapies.

## Supplementary Materials

The following are available online at https://www.mdpi.com/2077-0383/8/4/497/s1, Figure S1: Morphological and Immunophenotypic characterization of BMSCs.
