# Supplementary material for: Effect of Comedications and Endotoxins on Mesenchymal Stem Cell Secretomes, Migratory and Immunomodulatory Capacity
Source: J Clin Med. 2019 Apr 11;8(4):497. doi: 10.3390/jcm8040497 (PMC6517980; doi:10.3390/jcm8040497)
Supplement: Supplementary file 1 [file jcm-08-00497-s001.zip › jcm-474554-supplementary.docx]

| 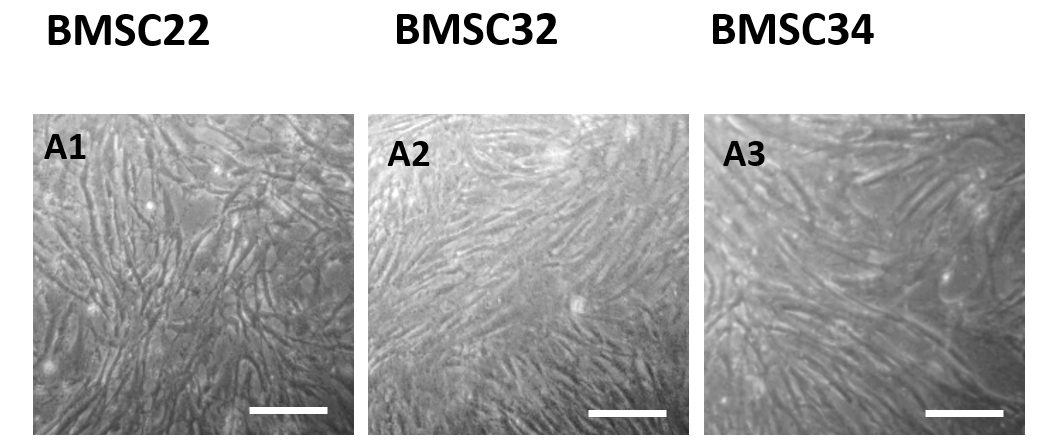 |
| --- |
| (**A**) |
| 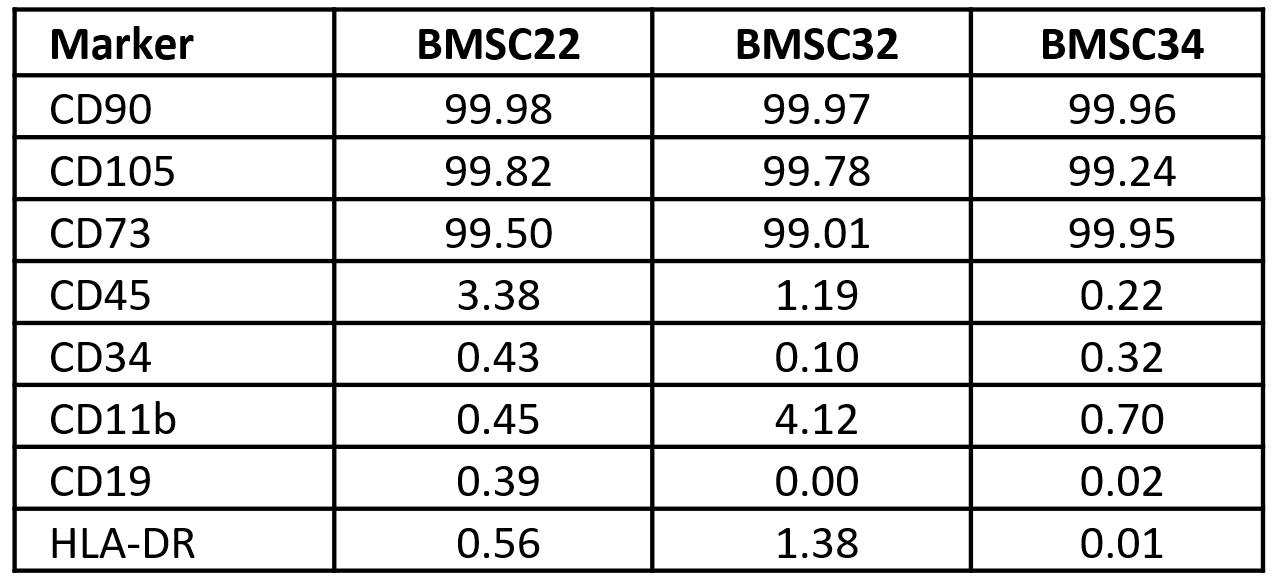 |
| (**B**) |

**Figure S1.** Morphological and Immunophenotypic characterization of bone marrow-derived MSCs (BMSCs). (**A**) Microscopic depictions of BMSCs (Scale bar is 50 µM, Magnification is 40×). (**B**) Percentage of surface marker expression determined by flow cytometric analysis.
